# Molecular epidemiology and clinical characteristics of the type VI secretion system in *Klebsiella pneumoniae* causing abscesses

**DOI:** 10.3389/fmicb.2023.1181701

**Published:** 2023-05-17

**Authors:** Peilin Liu, Awen Yang, Bin Tang, Zhiqian Wang, Zijuan Jian, Yanjun Liu, Jiahui Wang, Baiyun Zhong, Qun Yan, Wenen Liu

**Affiliations:** ^1^Department of Clinical Laboratory, Xiangya Hospital, Central South University, Changsha, Hunan, China; ^2^National Clinical Research Center for Geriatric Disorders, Xiangya Hospital, Changsha, Hunan, China

**Keywords:** type VI system (T6SS), hypervirulent *Klebsiella pneumoniae* (hvKP), abscess, hypervirulent, virulence

## Abstract

**Purpose:**

The type VI system (T6SS) has the potential to be a new virulence factor for hypervirulent *Klebsiella pneumoniae* (hvKp) strains. This study aimed to characterize the molecular and clinical features of T6SS-positive and T6SS-negative *K. pneumoniae* isolates that cause abscesses.

**Patients and methods:**

A total of 169 non-duplicate *K. pneumoniae* strains were isolated from patients with abscesses in a tertiary hospital in China from January 2018 to June 2022, and clinical data were collected. For all isolates, capsular serotypes, T6SS genes, virulence, and drug resistance genes, antimicrobial susceptibility testing, and biofilm formation assays were assessed. Multilocus sequence typing was used to analyze the genotypes of hvKp. T6SS-positive hvKp, T6SS-negative hvKp, T6SS-positive cKP, and T6SS-negative cKP (*n* = 4 strains for each group) were chosen for the *in vivo Galleria mellonella* infection model and *in vitro* competition experiments to further explore the microbiological characteristics of T6SS-positive *K. pneumoniae* isolates.

**Results:**

The positive detection rate for T6SS was 36.1%. The rates of hvKp, seven virulence genes, K1 capsular serotype, and ST23 in T6SS-positive strains were all higher than those in T6SS-negative strains (*p* < 0.05). Multivariate logistic regression analysis indicated that the carriage of aerobactin (OR 0.01) and *wcaG* (OR 33.53) were independent risk factors for T6SS-positive strains (*p* < 0.05). The T6SS-positive strains had a stronger biofilm-forming ability than T6SS-negative strains (*p* < 0.05). The T6SS-positive and T6SS-negative strains showed no significant differences in competitive ability (*p* = 0.06). In the *in vivo G. mellonella* infection model, the T6SS(+)/hvKP group had the worst prognosis. Except for cefazolin and tegacyclin, T6SS-positive isolates displayed a lower rate of antimicrobial resistance to other drugs (*p* < 0.05). The T6SS-positive isolates were more likely to be acquired from community infections (*p* < 0.05).

**Conclusion:**

*Klebsiella pneumoniae* isolates causing abscesses have a high prevalence of T6SS genes. T6SS-positive *K. pneumoniae* isolates are associated with virulence, and the T6SS genes may be involved in the hvKp virulence mechanism.

## 1. Introduction

*Klebsiella pneumoniae* is a threat to public health and can cause a range of severe infections, such as pneumonia, septicemia, meningitis, urinary tract infection, and bacterial abscesses (Dong et al., 2019; Lai and Yu, 2021). Based on its virulence features, *K. pneumoniae* is commonly classified into two groups: classic *K. pneumoniae* (cKp) and hypervirulent *K. pneumoniae* (hvKp) (Liu and Guo, 2018). CKp exhibits low virulence and with high resistance to antibiotics, which primarily causes pneumoniae, urinary tract infection and bacteremia in patients with severe underlying diseases or immunocompromised (Zhao et al., 2021; Kamau et al., 2022). Currently, hvKp has drawn extensive attention from researchers and clinicians, as it induces invasive abscess infections, especially liver abscesses, and has high pathogenicity resulting in high mortality (Zhu et al., 2021; Lin et al., 2022). Thus, knowledge of the underlying pathogenesis of hvKp is important for targeted disease prevention and control.

Protein secretion apparatuses play critical roles in bacterial survival and adaptation to complex environments and are used by bacteria to transport proteins through their cell membranes into external host cells (Morra et al., 2018). Currently, nine secretion systems are known: T1SS, T2SS, T3SS, T4SS, T5SS, T6SS, T7SS, T8SS, and T9SS (Hui et al., 2021). The type VI system (T6SS) is widespread in gram-negative bacteria (Yu et al., 2021), and in *K. pneumoniae*, it promotes bacterial competition, cell invasion, type-1 fimbriae expression, and *in vivo* colonization (Hsieh et al., 2019).

Strong iron uptake is one of the key features of hvKp (Choby et al., 2020; Nicolo et al., 2022). Genomic analysis identified that several genes coding for iron uptake are encoded around the T6SS gene; thus, T6SS could be involved in iron uptake in hvKp and may be a new virulence factor in hvKp strains (Barbosa and Lery, 2019; Zhu et al., 2021; Altayb et al., 2022). Two studies reported the frequency of the T6SS gene in *K. pneumoniae* isolated from bloodstream infections (Zhou et al., 2020; Zhang et al., 2022). However, the incidence rate of the T6SS gene in *K. pneumoniae* causing abscesses and *in vivo* studies of T6SS virulence has not been reported.

Here, we chose clinical *K. pneumoniae*-induced abscesses which usually have a high incidence of hvKp to obtain further insight into the T6SS relationships with hvKp. This study assessed the distribution of T6SS genes and characterized the molecular and clinical features of T6SS-positive and T6SS-negative isolates to further investigate the connection between T6SS and hypervirulence and to better understand the virulence mechanisms of hvKp.

## 2. Materials and methods

### 2.1. Isolates and clinical data collection

We isolated 169 non-duplicate *K. pneumoniae* strains from patients with abscesses in a tertiary hospital in China from January 2018 to June 2022 and stored them at −80°C. *K. pneumoniae* isolates were identified using matrix-assisted laser desorption/ionization-time of flight mass spectrometry (Bruker Daltonics). Abscess infections in which the detection of bacteria occurred in ≤48 h after admission were defined as community-acquired, while those that took >48 h after admission and infections associated with medical devices were classified as hospital-acquired (Anderson et al., 2017).

In addition, the following clinical information was collected from the patients’ electronic medical records: age, sex, acquisition of infection, personal history, underlying disease, antibiotic use, invasive operations, and clinical outcomes.

### 2.2. Hyperviscosity phenotype

The hyperviscosity phenotype was detected using the string test. The strains were cultured on blood agar plates, and then a single colony was gently picked with an inoculation loop. If a viscous string with a length > 5 mm could be pulled out, the strain was considered to have a hyperviscous phenotype. The tests were repeated ≥3 times.

### 2.3. Detection of T6SS, capsular serotyping, virulence, and drug resistance genes

The presence of T6SS, capsular serotyping, virulence, and drug resistance genes was detected by polymerase chain reaction (PCR), as previously described (Zhou et al., 2020). Genomic DNA of *K. pneumoniae* was extracted using the boiling method. The PCR products were electrophoresed on 1.0% agarose gel at 100 V for 40 min and visualized using a Syngene gel imager to determine the presence of these genes. In this study, strains positive for *p-rmpA, rmpA2*, and *aerobactin* were defined as hvKP, and those all positive for *IcmF*, *vgrG*, and *hcp* were designated as T6SS-positive strains. The primers used in this study are listed in the Supplementary material.

### 2.4. Whole-genome sequencing and multilocus sequence typing (MLST)

Whole-genome sequencing was used to identify virulence genes and sequence types (STs) in 70 hvKp isolates. Genomic DNA was extracted using the Ezup Column Bacteria Genomic DNA Purification Kit (Sangon Biotech, Shanghai, China). After sample qualification and library preparation, the sequencing library was sequenced on a DNBSEQ-T7 with the PE150 model at Bioyi Biotechnology Co., Ltd. (Wuhan, China). The data were assembled using Unicycler (v0.4.8) (Wick et al., 2017) and the whole genome was obtained. STs were identified using the MLST database.^1^ The 70 hvKp isolates were aligned to the reference genome, SGH10 *K. pneumoniae* (CP025080), using Snippy software (v4.6.0), and the core gene single nucleotide polymorphism sites were extracted. After removing the recombinant regions using the Gubbins software, phylogenetic trees using the maximum likelihood method were constructed using the FastTree software and uploaded to the iTOL website^2^ for annotation. The sequence data were deposited in the National Center for Biotechnology Information database with the accession numbers PRJNA940638. Using as a reference sequence.

### 2.5. Biofilm formation assay

The 0.5 McFarland bacterial suspensions were diluted (1:100), and 200 μL of the suspension was pipetted into a 96-well plate (three parallel wells were set up for each sample), and the microtiter plate was incubated for 24 h at 37°C. After pouring out the bacterial solution, each well was gently rinsed (3×) with 200 μL phosphate-buffered saline and air-dried. Subsequently, each well was stained with 200 μL of 1% crystalline violet for 15 min, the excess dye was washed in the wells and left to dry in the room, and 200 μL of 95% ethanol was added to each well to dissolve the crystal violet. After 10 min, the absorbance values were measured at 570 nm three times to obtain an average value. The mean OD_570_ value of the blank control plus 3× standard deviation was used as the ODc. Biofilm formation was defined as negative, weak, moderate, and strong if OD ≤ ODc, ODc < OD ≤ 2ODc, 2ODc < OD ≤ 4ODc, and 4ODc < OD, respectively.

### 2.6. *In vitro* competition assay

*In vitro* competition assays were performed as described previously, with slight modification (Zhang et al., 2022). *K. pneumoniae* isolates and *Escherichia coli* strain 25,922 were cultured overnight at 37°C in liquid Luria-Bertani (LB) medium. After standardization to an OD_600_ of 0.5, each culture was diluted (1:100) in 10 mL LB liquid medium. Suspensions of *K. pneumoniae* and *E. coli* were mixed at a ratio of 2:1 and incubated at 180 rpm and 37°C for 20 h. Serial 10-fold dilutions were applied to LB plates with or without ampicillin (50 μg/mL) and the cell counts of each strain were determined. The relative competitive index (CI) was calculated as the ratio of ampicillin-resistant *K. pneumoniae* to ampicillin-susceptible *E. coli*.

### 2.7. *In vivo Galleria mellonella* infection model

An *in vivo* virulence assay was performed using the *G. mellonella* infection model. Ten *G. mellonella* larvae weighing 250–350 mg were used for each strain (purchased from Tianjin Huiyude Biotechnology). Bacterial suspensions (10 μL; 1 × 10^6^ colony forming unit [CFU]) were inoculated into the left hind foot, and the number of deaths was recorded every 12 h and observed continuously for 72 h. All experiments were performed three times.

### 2.8. Antimicrobial susceptibility testing

Antimicrobial susceptibility testing was performed using the VITEK®2 Compact system (bioMerieux, Marcy l’Etoile, France) according to the manufacturer’s instructions. A panel of 20 antimicrobial agents was identified on the gram-negative Bacillus drug sensitivity identification card. *K. pneumoniae* ATCC700603 and *E. coli* ATCC25922 were used as the quality control strains. Bacterial resistance was determined based on minimal inhibitory concentrations (MIC) according to the Clinical and Laboratory Standards Institute 2022 standard (Lewis, 2022).

### 2.9. Statistical analysis

Categorical variables were analyzed using the chi-squared test or Fisher’s exact probability method. Student’s *t*-test or Mann–Whitney *U*-test was used to analyze numerical variables. *p*-value <0.05 was considered statistically significant. Virulence, resistance, and capsular serotyping genes, and clinical characteristics were analyzed by one-way logistic regression and multi-way logistic regression to determine risk factors for T6SS-positive *K. pneumoniae* infection of abscess origin. All data analyses were performed using the R4.2.1.

### 2.10. Ethics statement

Approval to collect patient medical records (which were anonymized) and *K. pneumoniae* strains was granted by the Ethics Committee of Xiangya Hospital, Central South University. Informed consent was not obtained due to the retrospective nature of the study. This study was also approved by the Central South University Ethics Committee (ID 202212281; Changsha, Hunan Province, People’s Republic of China).

## 3. Results

### 3.1. Clinical characteristics

The average age of all patients was 52.41 (46.00–63.00) years, and 71.01% (120/169) were men (Table 1); there were no obvious differences in age or sex between the two groups. The most common abscess site was the liver (33.14%), followed by the skin (12.43%). The most common disease was diabetes (33.73%), followed by liver and gallbladder diseases (32.54%), and lung diseases (31.95%). Most patients had antibiotic exposure before diagnosis (83.43%), and a few patients had multiple bacterial infections (37.28%). Among the T6SS-positive patients, the prevalence of hyperlipidemia was high (*p* < 0.05). Moreover, there were more community infections in the T6SS-positive group than in the T6SS-negative group (p < 0.05), and there were no significant differences in age, sex and other clinical characteristics (*p* > 0.05).

**Table 1:** Clinical characteristics of type VI secretion system (T6SS)-positive and T6SS-negative *Klebsiella pneumoniae* isolates causing abscesses.

Characteristics	ALL *N* = 169 (%)	T6SS-negative *N* = 108 (%)	T6SS-positive *N* = 61 (%)	*p*.overall
Age	52.41 (46.00–63.00)	51.00 (42.75–62.25)	55.00 (48.00–63.00)	0.153
Sex				0.089
Female	120 (71.01)	82 (75.93)	38 (62.30)	
Male	49 (28.99)	26 (24.07)	23 (37.70)	
Primary site				
Liver	56 (33.14)	30 (27.78)	26 (42.62)	0.072
Skin	21 (12.43)	17 (15.74)	4 (6.56)	0.135
Abdomen	19 (11.24)	10 (9.26)	9 (14.75)	0.405
Pelvic cavity	11 (6.51)	7 (6.48)	4 (6.56)	1.000
Peripancreatic	10 (5.92)	9 (8.33)	1 (1.64)	0.097
kidney	9 (5.33)	7 (6.48)	2 (3.28)	0.491
Others	43 (25.44)	28 (25.93)	15 (24.59)	0.994
Underlying condition				
Diabetes mellitus	57 (33.73)	36 (33.33)	21 (34.43)	1.000
Hepatobiliary diseases	55 (32.54)	37 (34.26)	18 (29.51)	0.644
Lung disease	54 (31.95)	39 (36.11)	15 (24.59)	0.170
Cancer	38 (22.49)	25 (23.15)	13 (21.31)	0.934
Hypertension	33 (19.53)	21 (19.44)	12 (19.67)	1.000
Septicemia	26 (15.38)	21 (19.44)	5 (8.20)	0.085
Hematological	23 (13.61)	12 (11.11)	11 (18.03)	0.305
Chronic kidney disease	19 (11.24)	12 (11.11)	7 (11.48)	1.000
Cardio-cerebrovascular disease	12 (7.10)	9 (8.33)	3 (4.92)	0.540
Hyperlipidemia	9 (5.33)	9 (8.33)	0 (0.00)	0.027
Personal history				
Surgery history	87 (51.48)	59 (54.63)	28 (45.90)	0.352
Smoking history	37 (21.89)	22 (20.37)	15 (24.59)	0.657
Drinking history	30 (17.75)	20 (18.52)	10 (16.39)	0.891
Trauma history	20 (11.83)	15 (13.89)	5 (8.20)	0.394
Radiotherapy or chemotherapy history	13 (7.69)	8 (7.41)	5 (8.20)	1.000
Blood transfusion history	10 (5.92)	8 (7.41)	2 (3.28)	0.332
Antibiotic exposure	141 (83.43)	93 (86.11)	48 (78.69)	0.302
Multiple bacterial infections	63 (37.28)	46 (42.59)	17 (27.87)	0.083
Acquisition				**0.037**
Hospital-acquired	110 (65.09)	77 (71.30)	33 (54.10)	
Community-acquired	59 (34.91)	31 (28.70)	28 (45.90)	

*p* < 0.05 was considered to be statistically significant. Statistically significant values are identified in boldface.

### 3.2. Distribution of T6SS, virulence- and drug-resistance genes, capsular serotypes, and hyperviscosity phenotype

Table 2 compares the distribution of hyperviscosity phenotype, virulence and drug resistance genes, and capsular types between T6SS-positive and T6SS-negative *K. pneumoniae* isolates. We tested the prevalence of 11 virulence genes: *rmpA*, *rmpA2*, aerobactin, *iroB*, *peg344*, *mrkD*, *wcaG*, *ybtS*, *alls*, *Kfu*, *entB*, and *iucA*. The positive rates of *rmpA*, *rmpA2*, aerobactin, *iroB*, *wcaG*, *all*, and *Kfu* were significantly higher in the T6SS-positive *K. pneumoniae* strains than in the T6SS-negative isolates (*p* < 0.05). In contrast, the positive rates of *K. pneumoniae* carbapenemase (KPC) and KPC-2 drug resistance genes in the T6SS-negative *K. pneumoniae* isolates were significantly higher than those in the T6SS-positive *K. pneumoniae* isolates (*p* < 0.001). As determined by *p-rmpA*, *iroB*, and *iucA*, 81 strains (47.65%) were identified as hvKp. Among T6SS-positive strains, the proportion of hvKp was significantly high (p < 0.001). Among the 169 *K. pneumoniae* strains, capsular types K1, K2, and K64 accounted for 25.88% (44/170), 21.18% (36/170), and 11.76% (20/170), respectively. T6SS-positive *K.pneumoniae* strains were observed to have a significant high prevalence for K1 serotype and T6SS-negative *K.pneumoniae* isolates have a significant high prevalence for K2 serotype (*p* < 0.001).

**Table 2:** Hyperviscosity phenotype, virulence, and drug resistance genes and capsular serotypes distribution of T6SS-positive and T6SS-negative *K. pneumoniae* isolates causing abscesses.

Hyperviscosity phenotype, virulence, and drug resistance genes and capsular serotypes	ALL *N = 169 (%)*	T6SS-negative *N = 108(%)*	T6SS-positive *N = 61 (%)*	*p*.overall
Hyperviscosity phenotype	81 (47.93)	53 (49.07)	28 (45.90)	0.813
HvKp	70 (41.42)	32 (29.63)	38 (62.30)	<0.001*
Virulence genes				
rmpA	100 (59.17)	54 (50.00)	46 (75.41)	0.002*
rmpA2	81 (47.93)	41 (37.96)	40 (65.57)	0.001*
aerobactin	96 (56.80)	54 (50.00)	42 (68.85)	0.027*
iroB	98 (57.99)	55 (50.93)	43 (70.49)	0.021*
peg344	108 (63.91)	66 (61.11)	42 (68.85)	0.401
mrkD	169 (100.00)	108 (100.00)	61 (100.00)	1.000
wcaG	46 (27.22)	5 (4.63)	41 (67.21)	<0.001*
ybts	119(70.41)	72 (66.67)	47 (77.05)	0.213
alls	50 (29.59)	9 (8.33)	41 (67.21)	<0.001*
kfu	58 (34.32)	20 (18.52)	38 (62.30)	<0.001*
entB	154 (91.12)	99 (91.67)	55 (90.16)	0.961
iutA	101 (59.76)	58 (53.70)	43 (70.49)	**0.048**
Drug resistance genes				
NDM-1	1 (0.59)	1 (0.93)	0 (0.00)	1.000
OXA-48	2 (1.18)	1 (0.93)	1 (1.64)	1.000
TEM	42 (24.85)	29 (26.85)	13 (21.31)	0.538
KPC-2	26 (15.38)	26 (24.07)	0 (0.00)	<0.001*
KPC	26 (15.38)	26 (24.07)	0 (0.00)	<0.001*
CTX-M	19 (11.24)	9 (8.26)	10 (16.39)	0.180
IMP	0 (0)	0 (0)	0 (0)	-
VIM	0 (0)	0 (0)	0 (0)	-
SHV	169 (100.00)	109 (100.00)	61 (100.00)	1.000
Capsular serotypes				
K1	44 (26.04)	4 (3.70)	40 (65.57)	**<0.001**
K2	36 (21.30)	35 (32.41)	1 (1.64)	**<0.001**
K5	2 (1.18)	2 (1.86)	0 (0.00)	0.537
K20	3 (1.78)	3 (2.78)	0 (0.00)	0.554
K54	2 (1.18)	2 (1.85)	0 (0.00)	0.537
K57	5 (2.96)	3 (2.78)	2 (3.28)	1.000
K64	19 (11.24)	19 (17.59)	0 (0.00)	**0.001**

*p* < 0.05 was considered to be statistically significant. Statistically significant values are identified in boldface.

### 3.3. MLST and phylogenic analysis

**Figure 1:**
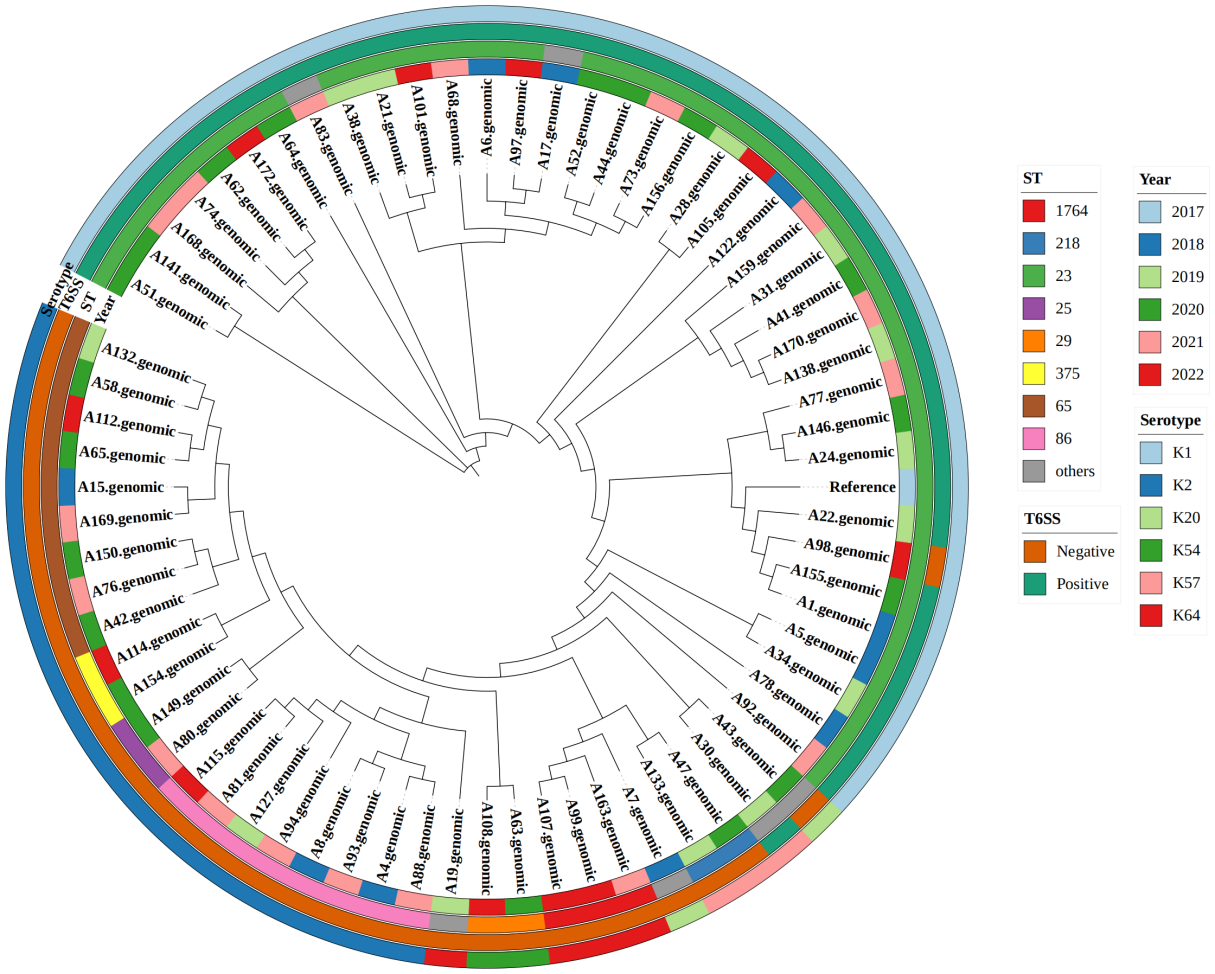
Phylogenetic tree of 70 hypervirulent *Klebsiella pneumoniae* isolates. From outside to center, rings 1, 2, 3, and 4 show the serotype, T6SS gene, year, and sequence type, respectively.

As shown in Table 3 and the core genome phylogenetic tree (Figure 1), similar to other studies, the most common ST in hvKp was ST23 (51.43%), and the most common serotype in hvKp was K1 (54.29%). Among the 36 ST23 strains, 16 had K1 capsules. From 2018 to 2022, there were 10,13,19, 17, and 11 hvKp strains. In T6SS-positive hvKp, the positivity rate of ST23 was 92.11%, which was higher than that of T6SS-negative hvKp (3.12%). In addition, the positive rates of ST65 and ST86 in T6SS-positive hvKp were far lower than that in T6SS-negative hvKp.

**Table 3:** Multilocus sequence typing of T6SS-positive and T6SS-negative *K. pneumoniae* isolates causing abscesses.

Sequence types	ALL *N =* 70 *(%)*	T6SS-negative *N = 32 (%)*	T6SS-positive *N = 38 (%)*	*p*.overall
ST23	36 (51.43)	1 (3.12)	35 (92.11)	**<0.001**
ST65	9 (12.86)	9 (28.12)	0 (0.00)	**<0.001**
ST86	8 (11.43)	8 (25.00)	0 (0.00)	**0.001**
ST25	2 (2.86)	2 (6.25)	0 (0.00)	0.205
ST29	2 (2.86)	2 (6.25)	0 (0.00)	0.205
ST218	2 (2.86)	2 (6.25)	0 (0.00)	0.205
ST375	2 (2.86)	2 (6.25)	0 (0.00)	0.205
ST1764	3 (4.29)	3 (9.38)	0 (0.00)	0.091
Others	6 (8.57)	3 (9.38)	3 (7.89)	1.000

*p* < 0.05 was considered to be statistically significant. Statistically significant values are identified in boldface.

### 3.4. Antimicrobial susceptibility

Among the 169 *K. pneumoniae* isolates, in addition to natural resistance to ampicillin, *K. pneumoniae* had the highest resistance to cefazolin (36.09%), and the resistance rate to tigecycline was as low as 1.78% (Table 4). The drug resistance of all T6SS-positive *K. pneumoniae* was lower than that of the T6SS-negative strains. Except for cefazolin and tigecyline, the drug sensitivity results for the other drugs were significantly different (*p* < 0.05). In addition, *K. pneumoniae* with K1 serotype showed lower drug resistance to 19 drugs except for ampicillin (*p* < 0.05) and *K. pneumoniae* with K2 and K57 serotype showed lower drug resistance to 10 drugs and 8 drug, respectively (*p* < 0.05). In contrast, *K. pneumoniae* with K64 serotype showed higher drug resistance to 18 drugs except for ampicillin and tigecycline (*p* < 0.05). The detailed results were given in the Supplementary material.

**Table 4:** Antimicrobial resistance of T6SS-positive and T6SS-negative *K. pneumoniae* isolates causing abscesses.

Antimicrobial agent	ALL *N = 169 (%)*	T6SS-negative *N = 108 (%)*	T6SS-positive *N = 61 (%)*	*p*.overall
Ampicillin	169 (100.00%)	108 (100.00%)	61 (100.00%)	1.000
Piperacillin/ Tazobactam	33 (19.53%)	32 (29.63%)	1 (1.64%)	**<0.001**
Ampicillin/Sulbactam	56 (33.14%)	45 (41.67%)	11 (18.03%)	**0.003**
Cefoperazone/Sulbactam	34 (20.12%)	31 (28.70%)	3 (4.92%)	**<0.001**
Cefazolin	61 (36.09%)	46 (42.59%)	15 (24.59%)	0.102
Cefuroxime	58 (34.32%)	44 (40.74%)	14 (22.95%)	**0.042**
Cefatriaxone	57 (33.73%)	44 (40.74%)	13 (21.31%)	**0.010**
Cefepime	38 (22.49%)	34 (31.48%)	4 (6.56%)	**<0.001**
Cefotetan	39 (23.08%)	32 (29.63%)	7 (11.48%)	**0.008**
Amtreonam	49 (28.99%)	39 (36.11%)	10 (16.39%)	**0.011**
Ertapenem	30 (17.75%)	29 (26.85%)	1 (1.64%)	**<0.001**
Meropenem	31 (18.34%)	30 (27.78%)	1 (1.64%)	**<0.001**
Imipenem	31 (18.34%)	30 (27.78%)	1 (1.64%)	**<0.001**
Gentamicin	30 (17.75%)	27 (25.00%)	3 (4.92%)	**0.001**
Amikacin	21 (12.43%)	20 (18.52%)	1 (1.64%)	**<0.001**
Tobramycin	26 (15.38%)	24 (22.22%)	2 (3.28%)	**<0.001**
Levofloxacin	40 (23.67%)	35 (32.41%)	5 (8.20%)	**<0.001**
Ciprofloxacin	55 (32.54%)	44 (40.74%)	11 (18.03%)	**0.004**
Sulfamethoxazole	43 (25.44%)	34 (31.48%)	9 (14.75%)	**0.016**
Tigecycline	3 (1.78%)	2 (1.85%)	1 (1.64%)	0.314

*p* < 0.05 was considered to be statistically significant. Statistically significant values are identified in boldface.

### 3.5. Risk factor analysis

**Figure 2:**
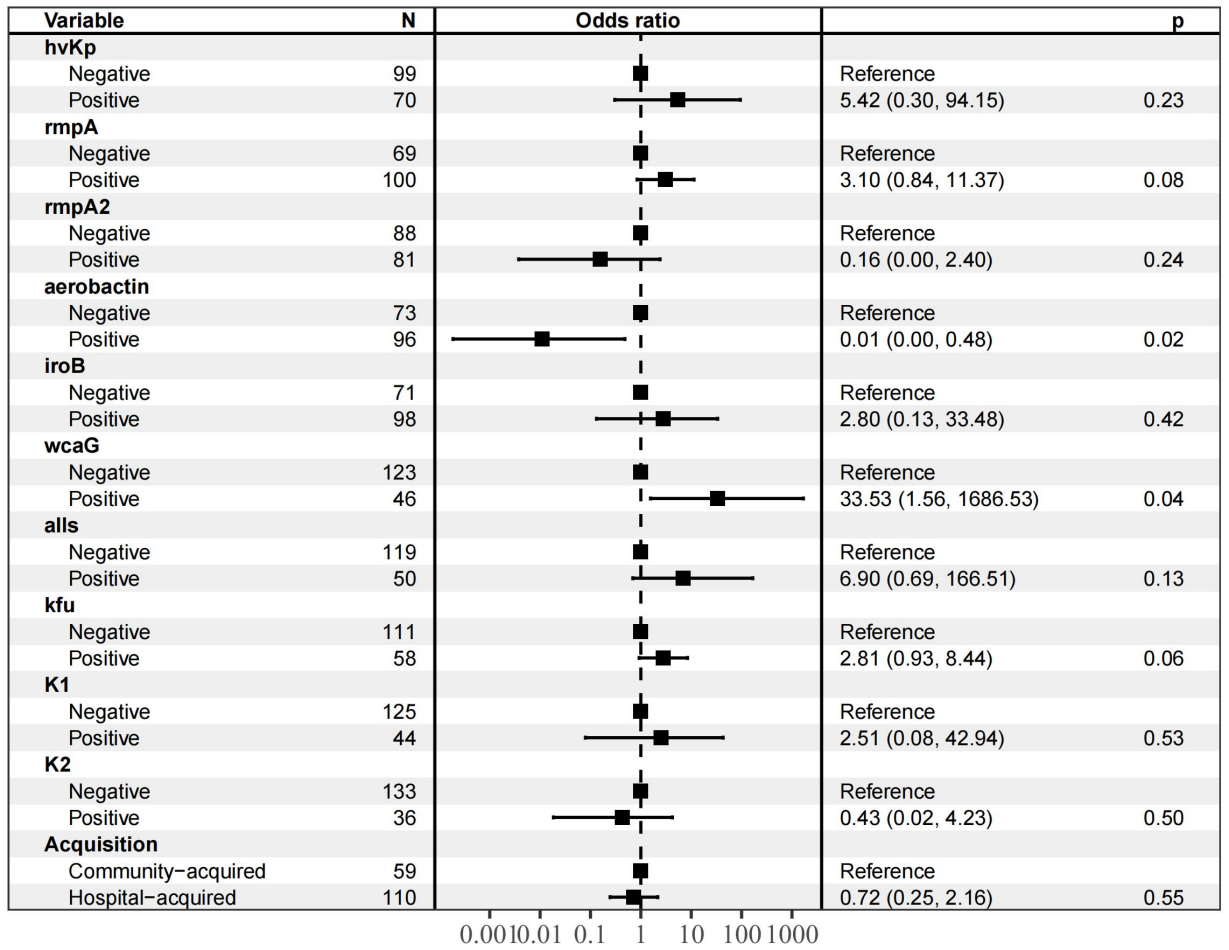
Multivariate analysis of prognostic factors for patients with T6SS − positive and T6SS-negative *K.pneumoniae* abscesses infections. The nine variables (hvKp, *rmpA*, *rmpA2*, aerobactin, *iroB*, *wcaG*, *alls*, *Kfu*, K1, K2 and acquisiyion) were included in multi-factor logistic regression, and aerobactin (OR 0.01) and WcaG (OR = 33.53)are independent risk factor for T6SS-positive strains infection (*p* < 0.05).

After single-factor logistic regression of virulence genes, drug resistance genes and clinical characteristics were included, and the results showed that the following nine genes were significantly related to T6SS-positive strains infection: *rmpA*, *rmpA2*, aerobactin, *iroB*, *wcaG*, *all*, *Kfu*, K1 and K2. In addition, hvKp and community-acquired infections were significantly associated with T6SS-positive strain infection. The above nine variables were included in multi-factor logistic regression (Figure 2); aerobactin(OR 0.01) and WcaG (OR = 33.53)are independent risk factor for T6SS-positive strains infection (*p* < 0.05).

### 3.6. Biofilm formation

**Figure 3:**
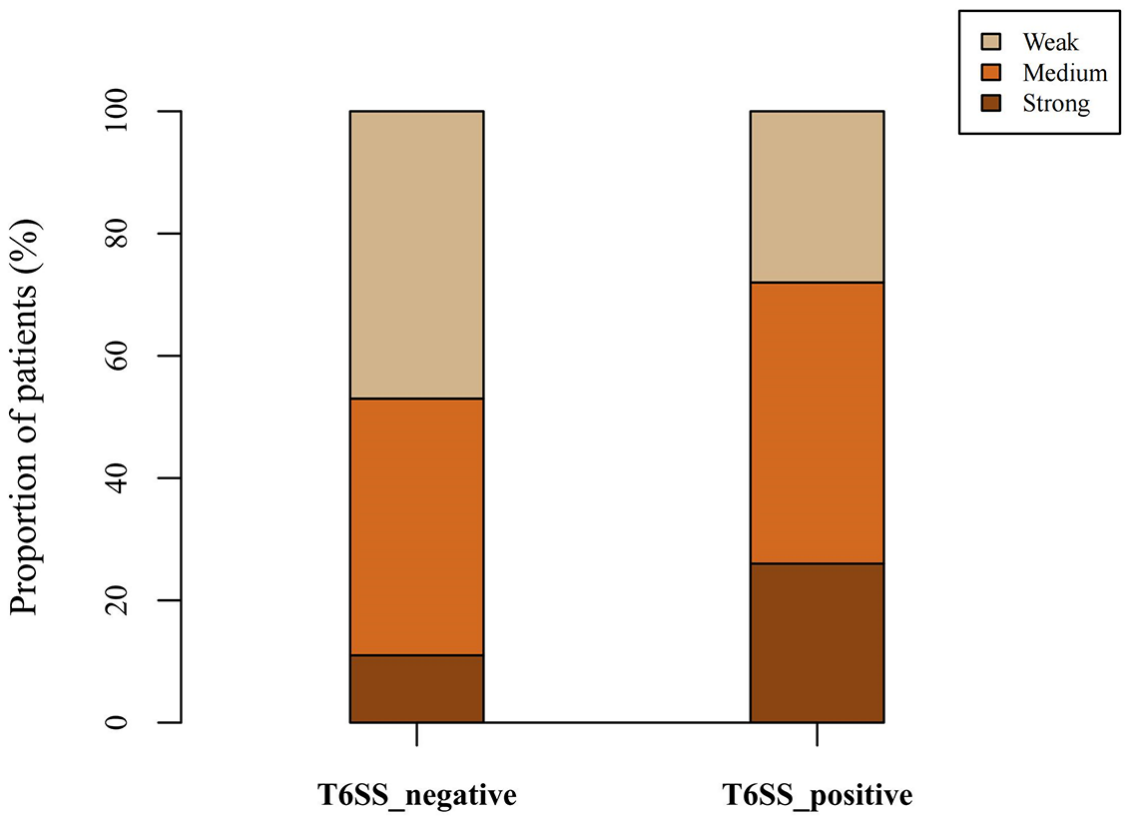
Proportion of biofilm-forming ability of T6SS-positive and T6SS-negative *K. pneumoniae* isolates causing abscesses. The proportion of isolates with strong biofilm-forming ability among T6SS-positive strains was higher than that of T6SS-negative strains.

Twenty-eight (16.57%), 74 (43.79%), and 67 (39.64%) strains had strong, medium, and weak biofilm-forming ability, respectively. As shown in Table 5; Figure 3, the proportion of isolates with strong biofilm-forming ability among T6SS-positive strains was higher than that of T6SS-negative strains (*p* < 0.05). Also, in different serotypes of *K. pneumoniae* strains only K64’s biofilm formation was statistically significant, and it had lower biofilm formation (*p* < 0.05). The detailed results were given in the Supplementary material.

**Table 5:** Biofilm formation ability of T6SS-positive and T6SS-negative *K. pneumoniae* isolates causing abscesses.

	ALL *N = 169 (%)*	T6SS-negative *N = 108 (%)*	T6SS-positive *N = 61 (%)*	*p*.overall
Result				**0.010**
Strong	28 (16.57)	12 (11.11)	16 (26.23)	
Medium	74 (43.79)	46 (42.59)	28 (45.90)	
Weak	67 (39.64)	50 (46.30)	17 (27.87)	

*p* < 0.05 was considered to be statistically significant. Statistically significant values are identified in boldface.

### 3.7. *In vitro* competition experiments

**Figure 4:**
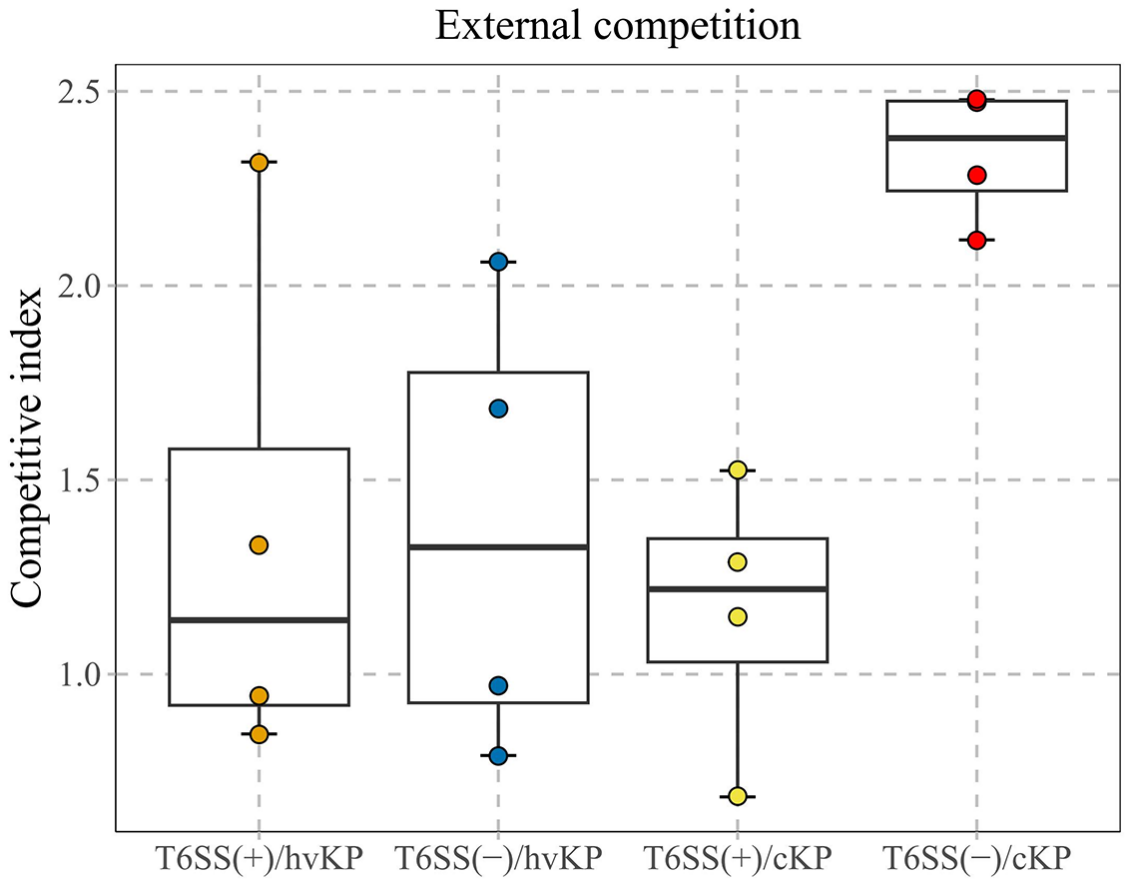
*ln vitro* competitive index (CI) values of T6SS *K. pneumoniae* isolates causing abscesses. Each circle represents one of the Cl values obtained from the number of ampicillin-resistant CFUs (*K. pneumoniae*) divided by the number of ampicillin-susceptible CFUs (*E. coli*). There was no difference between the competitiveness of T6SS-positive and T6SS-negative strains (*p* = 0.06), and there was no difference between the competitiveness of cKP and hvKP strains (*p* = 0.25).

This study calculated the CI values of 16 *K. pneumoniae* strains which were divided into four groups of four T6SS-positive and hvKp (T6SS[+]/hvKP), T6SS-positive and cKP (T6SS[+]/cKP), T6SS-negative and hvKp (T6SS[−]/hvKP), and T6SS-negative and cKP (T6SS[−]/cKP) strains (Figure 4). The average CI values of the T6SS(+)/hvKP, T6SS(+)/cKP, T6SS(−)/hvKP, and T6SS(−)/cKP were 1.36, 1.16, 1.38, and 2.34, respectively. The results showed that during co-culture with *E. coli*, there was no difference between the competitiveness of T6SS-positive and T6SS-negative strains (*p* = 0.06), and there was no difference between the competitiveness of cKP and hvKP strains (*p* = 0.25).

### 3.8. *In vivo* survival analysis of *Galleria mellonella* infection model

**Figure 5:**
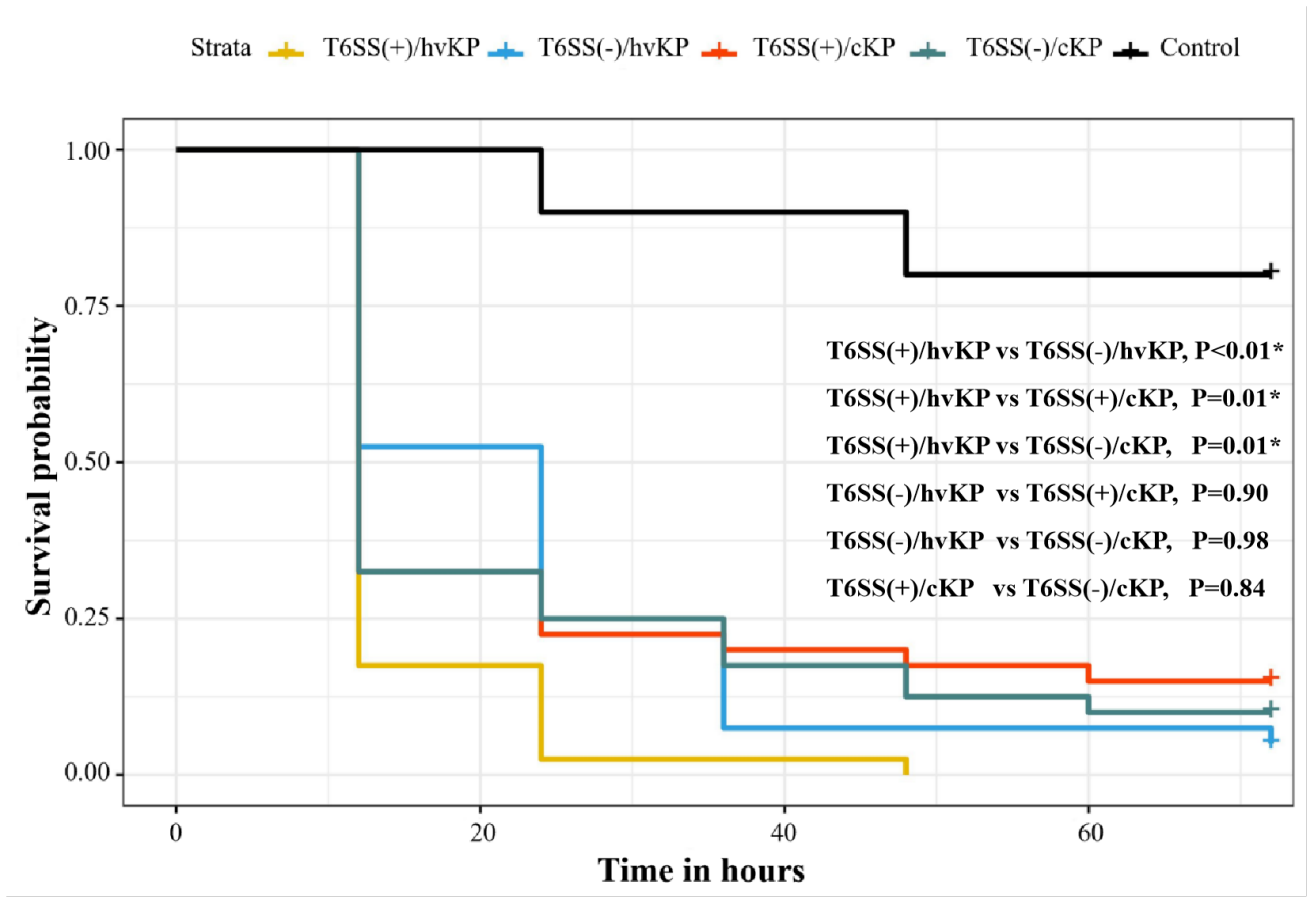
Evaluation of the virulence of *K. pneumoniae* isolates causing abscesses. Using a *G. mellonella* infection model, we investigated the virulence of 1 × 10^6^  CFU of each group of T6SS(+)/hvKP, T6SS(−)/hvKP, T6SS(+)/cKP, and T6SS(−)/cKP strains. Normal saline was used as control. The T6SS(+)/hvKP group had the worst prognosis, and the survival rate of this group was significantly different from the other three groups (*p* < 0.05).

We selected 16 *K. pneumoniae* strains for further analysis using an *in vivo G. mellonella* infection model and assessed the different groups as described above (section 3.7). The T6SS(+)/hvKP group had the worst prognosis, and the survival rate of this group was significantly different from the other three groups (*p* < 0.05; Figure 5).

## 4. Discussion

This study analyzed the molecular epidemiological characteristics of T6SS system genes in *K. pneumoniae* causing abscesses and the clinical features of 169 patients from January 2018 to June 2022. Studies that specifically investigate invasive abscess infections of *K. pneumoniae* isolates are rare.

Positive results for *hcp*, *icmF*, and *vgrG* genes confirmed T6SS positivity (Zhou et al., 2020; Liao et al., 2022; Zhang et al., 2022), and 36.1% (61/169) of our strains were T6SS-positive; this was much higher than the rate in blood stream infection (BSI) (20.1%) (Zhou et al., 2020). We identified hvKp in 41.42% (70/169) of patients and hypermucoviscosity phenotype in 47.93% (81/169). Moreover, the results indicated that there were more hvKp strains among T6SS-positive strains than among T6SS-negative strains (*p* < 0.001), which was similar to the findings of a study by Yin et al. (Zhang et al., 2022). The most prevalent hvKp isolates were ST23 (36/70), and ST23 in T6SS-positive isolates was significantly higher than in the T6SS-negative isolates. ST23 is closely related to high virulence, and *K. pneumoniae* ST23 is considered an important pathogen that causes hospital-acquired infections in critical patients (Lee et al., 2017; Hernandez et al., 2021; Du et al., 2022; Fan et al., 2022). These results suggest that the T6SS-positive *K. pneumoniae* strains are highly virulent.

However, there was no significant difference in the hypermucoviscosity phenotype between the T6SS-positive and T6SS-negative isolates. Previous studies have traditionally used the hypermucoviscosity phenotype to identify hvKp (Zhang et al., 2016). But increasing evidence has shown that hvKp may not be fully judged by hypermucoviscosity, and it is only one of the phenotypes that hvKp possesses (Liu and Guo, 2019; Su et al., 2020; Walker et al., 2020). Not all hvKp strains possessed the hypermucoviscosity phenotype in our study, and vice versa.

We investigated 11 virulence-associated and nine drug resistance-associated genes and found that the positive rates of *rmpA*, *rmpA2*, aerobactin, *iroB*, *wcaG*, *alls*, and *Kfu* were relatively higher for T6SS-positive isolates than for T6SS-negative isolates (*p* < 0.001). These seven virulence factors are associated with high pathogenicity: *rmpA* and *rmpA2* are involved in the hypermucoviscosity phenotype and capsular polysaccharide synthesis; aerobactin and *iroB* are siderophores, and aerobactin plays a critical role in hvKp virulence; and *wcaG* regulates fucose biosynthesis and enhances the ability of *K. pneumoniae* to resist neutrophil phagocytosis (Zheng et al., 2018; Russo and Gulick, 2019; Liu et al., 2022; Xu et al., 2022). These results further indicate that the T6SS-positive isolates were hypervirulent; however, the specific virulence mechanism involved in this process requires further study.

The T6SS-negative isolates displayed a higher rate of drug-resistant genes, KPC-2 and KPC, than T6SS-positive isolates (*p* < 0.001). KPC is a carbapenemase gene, and the presence of it could contribute the resistance to carbapenems (ertapenem, imipenem and meropenem) in *K. pneumoniae* (Effah et al., 2020; Cienfuegos-Gallet et al., 2022). Compared to the T6SS-positive isolates that all without KPC or KPC-2 gene, 24.07%(26/108) of T6SS-negative isolates had KPC and KPC-2 gene, and these T6SS-negative isolates all showed resistance to ertapenem, imipenem and meropenem. Antimicrobial susceptibility analysis revealed that the majority of T6SS-positive strains were sensitive to all antimicrobials except for ampicillin, similar to hvKp strains that are usually sensitive to antibiotics (Gu et al., 2018). Antibiotic resistance is often associated with decreased virulence (Mei et al., 2017). Hence, to a certain degree, this result shows that the T6SS genes may be linked to virulence.

K1 and K2 were strongly associated with high virulence in the previous study, and our results also found that K1 and K2 showed lower drug resistance (Lee et al., 2017; Sohrabi et al., 2022). In our study, T6SS-positive strains had a higher detection rate of the K1 capsular serotype than T6SS-negative strains. However, unlike other studies, the detection rates of K2 and K64 capsular serotypes in T6SS-negative strains were significantly higher than those of T6SS-positive strains (*p* < 0.05). Although little has been studied about the strength and weakness of the virulence of K1 and K2 serotypes, some studies have shown that K1 is more predominant in hvKp than K2 (Liu et al., 2014; Liu and Guo, 2018; Sohrabi et al., 2022). According to our result, it may also suggest that K1 were more likely associated with hvKp compared with K2. More studies are required to confirm this. In our study K64 showed higher drug resistance and it was reported that K64 was commonly associated with carbapenem resistance (Wang et al., 2022). The main mechanism of carbapenem resistance include enzyme production, efflux pumps and porin mutations (Suay-Garcia and Perez-Gracia, 2019). Our result found that K64 with higher drug resistance showed lower biofilm formation which suggested K64’s drug resistance mechanism probably not related to biofilm formation.

Patients infected with T6SS-positive isolates presented with clinical features similar to those of hvKp. Clinical characteristic analysis showed that community-acquired infections were more common in patients infected with T6SS-positive isolates than in those infected with T6SS-negative isolates. HvKp has often caused serious invasive community-acquired infections in previous studies (Wyres et al., 2020; Lam et al., 2021).

Our *in vivo* pathogenicity analysis in the *G. mellonella* infection model supported the above conclusion that larvae infected with T6SS-positive hvKp isolates showed a worse survival rate than the other groups (*p* < 0.05). Alternately, we found that the T6SS-negative hvKp isolate group had a worse survival rate than the T6SS-positive cKp isolates. This result might indicate that virulence genes were a larger contributor to survival rate than T6SS genes. However, this remains speculative, and no direct experimental results support this notion.

T6SS-positive isolates had stronger biofilm-forming abilities than T6SS-negative isolates. This was in agreement with the findings of Liao et al. but in contrast with Zhang et al. (2022) and Liao et al. (2022). T6SS genes are related to biofilm formation, but the role of T6SS has been controversial, and its specific mechanism needs further study (Chen et al., 2020; Dadashi et al., 2021; Fei et al., 2022).

In conclusion, this investigation showed the high virulence potential of T6SS-positive *K. pneumoniae* isolates, and T6SS genes may be involved in the hvKp virulence mechanism. The T6SS-positive isolates were more likely to be acquired from community infections. Most of the dominant virulence-associated factors (hvKp, K1, ST23, *rmpA*, *rmpA2*, and aerobactin) were highly clustered in T6SS-positive *K. pneumoniae* isolates and displayed lower rates of antimicrobial resistance, higher virulence in the *in vivo G. mellonella* infection model, and stronger biofilm abilities than T6SS-negative isolates. The limitations of this study are as follows: This was a single-center study; therefore, the molecular and clinical characteristics of *K. pneumoniae* isolates was regional. Multi-center studies would improve data reliability and further confirm our findings. Our future studies will aim to study the specific mechanism underlying how T6SS gene affects the virulence of *K. pneumoniae.*

## Data availability statement

The datasets presented in this study can be found in online repositories. The names of the repository/repositories and accession number(s) can be found in the article/Supplementary material.

## Author contributions

WL proposed and directed the study. PL, AY, and BT performed the experiments and collected the clinical data and prepared the first draft. AY and ZW analyzed the data. WL, PL, AY, BT, ZW, ZJ, YL, JW, BZ, and QY revised the manuscript. All authors contributed to the article and approved the submitted version.

## Funding

This work was supported by the National Natural Science Foundation of China (grant number 81672066) and National Key R&D Program of China (No. 2018YFC2000203).

## Conflict of interest

The authors declare that the research was conducted in the absence of any commercial or financial relationships that could be construed as a potential conflict of interest.

## Publisher’s note

All claims expressed in this article are solely those of the authors and do not necessarily represent those of their affiliated organizations, or those of the publisher, the editors and the reviewers. Any product that may be evaluated in this article, or claim that may be made by its manufacturer, is not guaranteed or endorsed by the publisher.

## Footnotes

^1^ https://bigsdb.pasteur.fr/

^2^ https://itol.embl.de/
