# Supplementary material for: Molecular epidemiology and clinical characteristics of the type VI secretion system in Klebsiella pneumoniae causing abscesses
Source: Front Microbiol. 2023 May 17;14:1181701. doi: 10.3389/fmicb.2023.1181701 (PMC10230222; doi:10.3389/fmicb.2023.1181701)
Supplement: Supplementary file 1 [file Table_1.DOCX]

Supplementary Material

**Molecular epidemiology and clinical characteristics of the type VI secretion system in *Klebsiella pneumoniae* causing abscesses**

Peilin Liu^1†^, Awen Yang^1†^, Bin Tang^1†^, Zhiqian Wang^1^, Zijuan Jian^1^, Yanjun Liu^1^, Jiahui Wang^1^, Baiyun Zhong^1^, Qun Yan^1^, ^1,2*^

*** Correspondence:** Wenen Liu [wenenliu@163.com](mailto:wenenliu@163.com)

# Supplementary Data

Table Primers used in this study.

| Primer name | DNA sequence(5’-3’) | Amplicon size (bp) | |
| --- | --- | --- | --- |
| T6SS genes | | | |
| *hcp* | F: TCCCGACCGATAACAACACC | 242 | |
|  | R: GATGTCGTGCATCAGGGGAT |  | |
| *vgrG* | F: TGAGCGTGTTTGTGCGAAAG | 259 | |
|  | R: TGACGCCCGTAATATCCTGC |  | |
| *icmF* | F: GACCGCTTACGGACAACTGA | 485 | |
|  | R: CACTCAGCACCCAGTCCATT |  | |
| Capsular serotypes | | |  |
| K1 | F: GGTGCTCTTTACATCATTGC | 1283 | |
|  | R: GCAATGGCCATTTGCGTTAG |  |  |
| K2 | F: GACCCGATATTCATACTTGACAGAG | 641 | |
|  | R: CCTGAAGTAAAATCGTAAATAGATGGC |  |  |
| K5 | F: TGGTAGTGATGCTCGCGA | 741 | |
|  | R: CCTGAACCCACCCCAATC |  |  |
| K20 | F: CGGTGCTACAGTGCATCATT | 280 | |
|  | R: GTTATACGATGCTCAGTCGC |  |  |
| K54 | F: CATTAGCTCAGTGGTTGGCT | 881 | |
|  | R: GCTTGACAAACACCATAGCAG |  |  |
| K57 | F: CTCAGGGCTAGAAGTGTCAT | 1037 | |
|  | R: CACTAACCCAGAAAGTCGAG |  |  |
| wzi | F:GTGCCGCGAGCGCTTTCTATCTTGGTATTCC | 831 | |
|  | R;GAGAGCCACTGGTTCCAGAACTTGACCGC |  | |
| Virulence genes | | | |
| *p-rmpA* | F:ACTGGGCTACCTCTGCTTCA | 535 | |
|  | R:CTTGCATGAGCCATCTTTCA |  | |
| *p-rmpA2* | F:GTGCAATAAGGATGTTACATTA | 430 | |
|  | R:GGATGCCCTCCTCCTG |  | |
| *Aerobactin* | F:GCATAGGCGGATACGAACAT | 556 | |
|  | R:CACAGGGCAATTGCTTACCT |  | |
| *iroB* | F:ATCTCATCATCTACCCTCCGCTC | 235 | |
|  | R:GGTTCGCCGTCGTTTTCAA |  | |
| *Peg-344* | F:CTTGAAACTATCCCTCCAGTC | 508 | |
|  | R:CCAGCGAAAGAATAACCCC |  | |
| *mrkD* | F:AAGCTATCGCTGTACTTCCGGCA | 340 | |
|  | R:GGCGTTGGCGCTCAGATAGG |  | |
| *wcaG* | F:GGTTGGKTCAGCAATCGTA | 169 | |
|  | R:ACTATTCCGCCAACTTTTGC |  | |
| *ybtS* | F:GACGGAAACAGCACGGTAAA | 242 | |
|  | R:GAGCATAATAAGGCGAAAGA |  | |
| *alls* | F:CATTACGCACCTTTGTCAGC | 764 | |
|  | R:GAATGTGTCGGCGATCAGCTT |  | |
| *kfu* | F:GGCCTTTGTCCAGAGCTACG | 638 | |
|  | R:GGGTCTGGCGCAGAGTATGC |  | |
| *entB* | F:GTCAACTGGGCCTTTGAGCCGTC | 400 | |
|  | R:TATGGGCGTAAACGCCGGTGAT |  | |
| *iutA* | F:GGGAAAGGCTTCTCTGCCAT | 920 | |
|  | R;TTATTCGCCACCACGCTCTT |  | |
| Drug-resistant genes | | | |
| *NDM-1* | F: GGGCAGTCGCTTCCAACGGT | 435 | |
|  | R: GTAGTGCTCAGTGTCGGCAT |  | |
| *OXA-48* | F: GCTTGATCGCCCTCGATT | 281 | |
|  | R: GATTTGCTCCGTGGCCGAAA |  | |
| *TEM* | F:AGTGCTGCCATAACCATGAGTG | 431 | |
|  | R:CTGACTCCCCGTCGTGTAGATA |  | |
| *KPC* | F: GCTACACCTAGCTCCACCTTC | 920 | |
|  | R: ACAGTGGTTGGTAATCCATGC |  | |
| *KPC-2* | F: CATTCAAGGGCTTTCTTGCTGC | 538 | |
|  | R: ACGACGGCATAGTCATTTGC |  | |
| *CTX-M* | F:TTGAGGCTGGGTGAAGT | 759 | |
|  | R:ACGCTGTTGTTAGGAAGTG |  | |
| *IMP* | F:TTGACACTCCATTTACTGCTA | 172 | |
|  | R:TCATTTGTTAATTCAGATGCATA |  | |
| *VIM* | F:GAGTTGCTTTTGATTGATACAG | 247 | |
|  | R:TCGATGAGAGTCCTTCTAGA |  | |
| *SHV* | F:GCCTTTATCGGCCTTCACTCAAG | 898 | |
|  | R:TTAGCGTTGCCAGTGCTCGATCA |  | |

Table . Antimicrobial resistance of K1-positive and K1-negative *K. pneumoniae* isolates causing abscesses

| **Antimicrobial agent** | **ALL**  ***N=169 (%)*** | **K1-negative**  ***N=125 (%)*** | **K1-positive**  ***N=44 (%)*** | **p.overall** |
| --- | --- | --- | --- | --- |
| Ampicillin | 169 (100.00) | 125 (100.00) | 44 (100.00) | 1.000 |
| Piperacillin/ Tazobactam | 33 (19.53) | 33 (26.40) | 0 (0.00) | **<0.001** |
| Ampicillin/Sulbactam | 56 (33.14) | 49 (39.20) | 7 (15.91) | **0.013** |
| Cefoperazone/Sulbactam | 34 (20.12) | 34 (27.20) | 0 (0.00) | **<0.001** |
| Cefazolin | 61 (36.09) | 53 (42.40) | 8 (18.18) | **0.011** |
| Cefuroxime | 58 (34.32) | 51 (40.80) | 7 (15.91) | **0.009** |
| Cefatriaxone | 57 (33.73) | 51 (40.80) | 6 (13.64) | **0.001** |
| Cefepime | 38 (22.49) | 37 (29.60) | 1 (2.27) | **<0.001** |
| Cefotetan | 39 (23.08) | 37 (29.60) | 2 (4.55) | **0.001** |
| Amtreonam | 49 (28.99) | 45 (36.00) | 4 (9.09) | **0.001** |
| Ertapenem | 30 (17.75) | 30 (24.00) | 0 (0.00) | **<0.001** |
| Meropenem | 31 (18.34) | 31 (24.80) | 0 (0.00) | **<0.001** |
| Imipenem | 31 (18.34) | 31 (24.80) | 0 (0.00) | **<0.001** |
| Gentamicin | 30 (17.75) | 29 (23.20) | 1 (2.27) | **0.001** |
| Amikacin | 21 (12.43) | 21 (16.80) | 0 (0.00) | **0.002** |
| Tobramycin | 26 (15.38) | 24 (19.20) | 2 (4.55) | **0.002** |
| Levofloxacin | 40 (23.67) | 39 (31.20) | 1 (2.27) | **<0.001** |
| Ciprofloxacin | 55 (32.54) | 51 (40.80) | 4 (9.09) | **<0.001** |
| Sulfamethoxazole | 43 (25.44) | 39 (31.20) | 4 (9.09) | **0.004** |
| Tigecycline | 3 (1.78) | 3 (2.40) | 0 (0.00) | 0.059 |

P<0.05 was considered to be statistically significant.

Table . Antimicrobial resistance of K2-positive and K2-negative *K. pneumoniae* isolates causing abscesses

| **Antimicrobial agent** | **ALL**  ***N=169 (%)*** | **K2-negative**  ***N=133(%)*** | **K2-positive**  ***N=36 (%)*** | **p.overall** |
| --- | --- | --- | --- | --- |
| Ampicillin | 169 (100.00) | 133 (100.00) | 36 (100.00) | 1.000 |
| Piperacillin/ Tazobactam | 33 (19.53) | 32 (24.06) | 1 (2.78) | **0.007** |
| Ampicillin/Sulbactam | 56 (33.14) | 52 (39.10) | 4 (11.11) | **0.001** |
| Cefoperazone/Sulbactam | 34 (20.12) | 31 (23.31) | 3 (8.33) | 0.160 |
| Cefazolin | 61 (36.09) | 58 (43.61) | 3 (8.33) | **<0.001** |
| Cefuroxime | 58 (34.32) | 54 (40.60) | 4 (11.11) | **<0.001** |
| Cefatriaxone | 57 (33.73) | 52 (39.10) | 5 (13.89) | **0.008** |
| Cefepime | 38 (22.49) | 34 (25.56) | 4 (11.11) | 0.176 |
| Cefotetan | 39 (23.08) | 36 (27.07) | 3 (8.33) | **0.035** |
| Amtreonam | 49 (28.99) | 46 (34.59) | 3 (8.33) | **0.002** |
| Ertapenem | 30 (17.75) | 29 (21.80) | 1 (2.78) | **0.006** |
| Meropenem | 31 (18.34) | 29 (21.80) | 2 (5.56) | 0.052 |
| Imipenem | 31 (18.34) | 29 (21.80) | 2 (5.56) | 0.052 |
| Gentamicin | 30 (17.75) | 27 (20.30) | 3 (8.33) | 0.281 |
| Amikacin | 21 (12.43) | 19 (14.29) | 2 (5.56) | 0.413 |
| Tobramycin | 26 (15.38) | 24 (18.05) | 2 (5.56) | 0.237 |
| Levofloxacin | 40 (23.67) | 37 (27.82) | 3 (8.33) | **0.021** |
| Ciprofloxacin | 55 (32.54) | 50 (37.59) | 5 (13.89) | **0.016** |
| Sulfamethoxazole | 43 (25.44) | 39 (29.32) | 4 (11.11) | 0.051 |
| Tigecycline | 3 (1.78) | 3 (2.26) | 0 (0.00) | 0.390 |

P<0.05 was considered to be statistically significant.

Table . Antimicrobial resistance of K5-positive and K5-negative *K. pneumoniae* isolates causing abscesses

| **Antimicrobial agent** | **ALL**  ***N=169 (%)*** | **K5-negative**  ***N=167(%)*** | **K5-positive**  ***N=2(%)*** | **p.overall** |
| --- | --- | --- | --- | --- |
| Ampicillin | 169 (100.00) | 167 (100.00) | 2 (100.00) | 1.000 |
| Piperacillin/ Tazobactam | 33 (19.53) | 33 (19.76) | 0 (0.00) | 1.000 |
| Ampicillin/Sulbactam | 56 (33.14) | 56 (33.53) | 0 (0.00) | 0.594 |
| Cefoperazone/Sulbactam | 34 (20.12) | 34 (20.36) | 0 (0.00) | 1.000 |
| Cefazolin | 61 (36.09) | 61 (36.53) | 0 (0.00) | 0.592 |
| Cefuroxime | 58 (34.32) | 58 (34.73) | 0 (0.00) | 0.223 |
| Cefatriaxone | 57 (33.73) | 57 (34.13) | 0 (0.00) | 0.558 |
| Cefepime | 38 (22.49) | 38 (22.75) | 0 (0.00) | 1.000 |
| Cefotetan | 39 (23.08) | 39 (23.35) | 0 (0.00) | 1.000 |
| Amtreonam | 49 (28.99) | 49 (29.34) | 0 (0.00) | 1.000 |
| Ertapenem | 30 (17.75) | 30 (17.96) | 0 (0.00) | 1.000 |
| Meropenem | 31 (18.34) | 31 (18.56) | 0 (0.00) | 1.000 |
| Imipenem | 31 (18.34) | 31 (18.56) | 0 (0.00) | 1.000 |
| Gentamicin | 30 (17.75) | 30 (17.96) | 0 (0.00) | 1.000 |
| Amikacin | 21 (12.43) | 21 (12.57) | 0 (0.00) | 1.000 |
| Tobramycin | 26 (15.38) | 26 (15.57) | 0 (0.00) | 1.000 |
| Levofloxacin | 40 (23.67) | 40 (23.95) | 0 (0.00) | 1.000 |
| Ciprofloxacin | 55 (32.54) | 55 (32.93) | 0 (0.00) | 0.585 |
| Sulfamethoxazole | 43 (25.44) | 42 (25.15) | 1 (50.00) | 0.454 |
| Tigecycline | 3 (1.78) | 3 (1.80) | 0 (0.00) | 0.275 |

Table . Antimicrobial resistance of K20-positive and K20-negative *K. pneumoniae* isolates causing abscesses

| **Antimicrobial agent** | **ALL**  ***N=169 (%)*** | **K20-negative**  ***N=166(%)*** | **K20-positive**  ***N=3(%)*** | **p.overall** |
| --- | --- | --- | --- | --- |
| Ampicillin | 169 (100.00) | 166 (100.00) | 3 (100.00) | 1.000 |
| Piperacillin/ Tazobactam | 33 (19.53) | 33 (19.88) | 0 (0.00) | 1.000 |
| Ampicillin/Sulbactam | 56 (33.14) | 56 (33.73) | 0 (0.00) | 0.628 |
| Cefoperazone/Sulbactam | 34 (20.12) | 34 (20.48) | 0 (0.00) | 1.000 |
| Cefazolin | 61 (36.09) | 61 (36.75) | 0 (0.00) | 0.435 |
| Cefuroxime | 58 (34.32) | 58 (34.94) | 0 (0.00) | 0.509 |
| Cefatriaxone | 57 (33.73) | 57 (34.34) | 0 (0.00) | 0.568 |
| Cefepime | 38 (22.49) | 38 (22.89) | 0 (0.00) | 1.000 |
| Cefotetan | 39 (23.08) | 39 (23.49) | 0 (0.00) | 0.682 |
| Amtreonam | 49 (28.99) | 49 (29.52) | 0 (0.00) | 0.572 |
| Ertapenem | 30 (17.75) | 30 (18.07) | 0 (0.00) | 1.000 |
| Meropenem | 31 (18.34) | 31 (18.67) | 0 (0.00) | 1.000 |
| Imipenem | 31 (18.34) | 31 (18.67) | 0 (0.00) | 1.000 |
| Gentamicin | 30 (17.75) | 30 (18.07) | 0 (0.00) | 1.000 |
| Amikacin | 21 (12.43) | 21 (12.65) | 0 (0.00) | 1.000 |
| Tobramycin | 26 (15.38) | 26 (15.66) | 0 (0.00) | 1.000 |
| Levofloxacin | 40 (23.67) | 40 (24.10) | 0 (0.00) | 0.657 |
| Ciprofloxacin | 55 (32.54) | 55 (33.13) | 0 (0.00) | 0.605 |
| Sulfamethoxazole | 43 (25.44) | 43 (25.90) | 0 (0.00) | 0.578 |
| Tigecycline | 3 (1.78) | 3 (1.81) | 0 (0.00) | 0.383 |

Table . Antimicrobial resistance of K54-positive and K54-negative *K. pneumoniae* isolates causing abscesses

| **Antimicrobial agent** | **ALL**  ***N=169 (%)*** | **K54-negative**  ***N=167(%)*** | **K54-positive**  ***N=2 (%)*** | **p.overall** |
| --- | --- | --- | --- | --- |
| Ampicillin | 169 (100.00) | 167 (100.00) | 2 (100.00) | 1.000 |
| Piperacillin/ Tazobactam | 33 (19.53) | 33 (19.76) | 0 (0.00) | **0.047** |
| Ampicillin/Sulbactam | 56 (33.14) | 55 (32.93) | 1 (50.00) | 1.000 |
| Cefoperazone/Sulbactam | 34 (20.12) | 34 (20.36) | 0 (0.00) | 1.000 |
| Cefazolin | 61 (36.09) | 60 (35.93) | 1 (50.00) | 1.000 |
| Cefuroxime | 58 (34.32) | 57 (34.13) | 1 (50.00) | 1.000 |
| Cefatriaxone | 57 (33.73) | 57 (34.13) | 0 (0.00) | 0.558 |
| Cefepime | 38 (22.49) | 38 (22.75) | 0 (0.00) | 1.000 |
| Cefotetan | 39 (23.08) | 39 (23.35) | 0 (0.00) | 1.000 |
| Amtreonam | 49 (28.99) | 49 (29.34) | 0 (0.00) | 1.000 |
| Ertapenem | 30 (17.75) | 30 (17.96) | 0 (0.00) | 1.000 |
| Meropenem | 31 (18.34) | 31 (18.56) | 0 (0.00) | 1.000 |
| Imipenem | 31 (18.34) | 31 (18.56) | 0 (0.00) | 1.000 |
| Gentamicin | 30 (17.75) | 30 (17.96) | 0 (0.00) | 1.000 |
| Amikacin | 21 (12.43) | 21 (12.57) | 0 (0.00) | 1.000 |
| Tobramycin | 26 (15.38) | 26 (15.57) | 0 (0.00) | 1.000 |
| Levofloxacin | 40 (23.67) | 40 (23.95) | 0 (0.00) | 1.000 |
| Ciprofloxacin | 55 (32.54) | 55 (32.93) | 0 (0.00) | 0.585 |
| Sulfamethoxazole | 43 (25.44) | 43 (25.75) | 0 (0.00) | 1.000 |
| Tigecycline | 3 (1.78) | 3 (1.80) | 0 (0.00) | 1.000 |

P<0.05 was considered to be statistically significant.

Table . Antimicrobial resistance of K57-positive and K57-negative *K. pneumoniae* isolates causing abscesses

| **Antimicrobial agent** | **ALL**  ***N=169 (%)*** | **K57-negative**  ***N=164(%)*** | **K57-positive**  ***N=5 (%)*** | **p.overall** |
| --- | --- | --- | --- | --- |
| Ampicillin | 169 (100.00) | 164 (100.00) | 5 (100.00) | 1.000 |
| Piperacillin/ Tazobactam | 33 (19.53) | 32 (19.51) | 1 (20.00) | **0.039** |
| Ampicillin/Sulbactam | 56 (33.14) | 55 (33.54) | 1 (20.00) | **0.049** |
| Cefoperazone/Sulbactam | 34 (20.12) | 33 (20.12) | 1 (20.00) | 0.050 |
| Cefazolin | 61 (36.09) | 60 (36.59) | 1 (20.00) | 0.124 |
| Cefuroxime | 58 (34.32) | 57 (34.76) | 1 (20.00) | 0.083 |
| Cefatriaxone | 57 (33.73) | 56 (34.15) | 1 (20.00) | 0.062 |
| Cefepime | 38 (22.49) | 37 (22.56) | 1 (20.00) | 0.068 |
| Cefotetan | 39 (23.08) | 38 (23.17) | 1 (20.00) | 0.459 |
| Amtreonam | 49 (28.99) | 48 (29.27) | 1 (20.00) | 0.083 |
| Ertapenem | 30 (17.75) | 29 (17.68) | 1 (20.00) | **0.036** |
| Meropenem | 31 (18.34) | 30 (18.29) | 1 (20.00) | **0.021** |
| Imipenem | 31 (18.34) | 30 (18.29) | 1 (20.00) | **0.021** |
| Gentamicin | 30 (17.75) | 29 (17.68) | 1 (20.00) | 1.000 |
| Amikacin | 21 (12.43) | 20 (12.20) | 1 (20.00) | **0.013** |
| Tobramycin | 26 (15.38) | 25 (15.24) | 1 (20.00) | **0.030** |
| Levofloxacin | 40 (23.67) | 39 (23.78) | 1 (20.00) | 0.384 |
| Ciprofloxacin | 55 (32.54) | 53 (32.32) | 2 (40.00) | 1.000 |
| Sulfamethoxazole | 43 (25.44) | 42 (25.61) | 1 (20.00) | **0.030** |
| Tigecycline | 3 (1.78) | 3 (1.83) | 0 (0.00) | 1.000 |

P<0.05 was considered to be statistically significant.

Table . Antimicrobial resistance of K64-positive and K64-negative *K. pneumoniae* isolates causing abscesses

| **Antimicrobial agent** | **ALL**  ***N=169 (%)*** | **K64-negative**  ***N=150(%)*** | **K64-positive**  ***N=19 (%)*** | **p.overall** |
| --- | --- | --- | --- | --- |
| Ampicillin | 169 (100.00) | 150 (100.00) | 19 (100.00) | 1.000 |
| Piperacillin/ Tazobactam | 33 (19.53) | 17 (11.33) | 16 (84.21) | **<0.001** |
| Ampicillin/Sulbactam | 56 (33.14) | 40 (26.67) | 16 (84.21) | **<0.001** |
| Cefoperazone/Sulbactam | 34 (20.12) | 20 (13.33) | 14 (73.68) | **<0.001** |
| Cefazolin | 61 (36.09) | 45 (30.00) | 16 (84.21) | **<0.001** |
| Cefuroxime | 58 (34.32) | 42 (28.00) | 16 (84.21) | **<0.001** |
| Cefatriaxone | 57 (33.73) | 41 (27.33) | 16 (84.21) | **<0.001** |
| Cefepime | 38 (22.49) | 22 (14.67) | 16 (84.21) | **<0.001** |
| Cefotetan | 39 (23.08) | 23 (15.33) | 16 (84.21) | **<0.001** |
| Amtreonam | 49 (28.99) | 33 (22.00) | 16 (84.21) | **<0.001** |
| Ertapenem | 30 (17.75) | 14 (9.33) | 16 (84.21) | **<0.001** |
| Meropenem | 31 (18.34) | 15 (10.00) | 16 (84.21) | **<0.001** |
| Imipenem | 31 (18.34) | 15 (10.00) | 16 (84.21) | **<0.001** |
| Gentamicin | 30 (17.75) | 19 (12.67) | 11 (57.89) | **<0.001** |
| Amikacin | 21 (12.43) | 10 (6.67) | 11 (57.89) | **<0.001** |
| Tobramycin | 26 (15.38) | 14 (9.33) | 12 (63.16) | **<0.001** |
| Levofloxacin | 40 (23.67) | 25 (16.67) | 15 (78.95) | **<0.001** |
| Ciprofloxacin | 55 (32.54) | 39 (26.00) | 16 (84.21) | **<0.001** |
| Sulfamethoxazole | 43 (25.44) | 33 (22.00) | 10 (52.63) | **0.018** |
| Tigecycline | 3 (1.78) | 2 (1.33) | 1 (5.26) | 0.188 |

P<0.05 was considered to be statistically significant.

Table . Biofilm formation ability of K1-positive and K1-negative *K. pneumoniae* isolates causing abscesses.

|  | **ALL**  ***N=169 (%)*** | **K1-negative**  ***N=125 (%)*** | **K1-positive**  ***N=44 (%)*** | **p.overall** |
| --- | --- | --- | --- | --- |
| result |  |  |  | 0.059 |
| Strong | 28 (16.57 ) | 16 (12.80%) | 12 (27.27%) |  |
| Medium | 74 (43.79 ) | 55 (44.00%) | 19 (43.18%) |  |
| Weak | 67 (39.64 ) | 54 (43.20%) | 13 (29.55%) |  |

**Table .** Biofilm formation ability of K2-positive and K2-negative *K. pneumoniae* isolates causing abscesses.

|  | **ALL**  ***N=169 (%)*** | **K2-negative**  ***N=133 (%)*** | **K2-positive**  ***N=36 (%)*** | **p.overall** |
| --- | --- | --- | --- | --- |
| result |  |  |  | 0.363 |
| Strong | 28 (16.57 ) | 24 (18.05%) | 4 (11.11%) |  |
| Medium | 74 (43.79 ) | 60 (45.11%) | 14 (38.89%) |  |
| Weak | 67 (39.64 ) | 49 (36.84%) | 18 (50.00%) |  |

Table . Biofilm formation ability of K5-positive and K5-negative *K. pneumoniae* isolates causing abscesses.

|  | **ALL**  ***N=169 (%)*** | **K5-negative**  ***N=167 (%)*** | **K5-positive**  ***N=2 (%)*** | **p.overall** |
| --- | --- | --- | --- | --- |
| result |  |  |  | 1.000 |
| Strong | 28 (16.57 ) | 28 (16.77%) | 0 (0.00%) |  |
| Medium | 74 (43.79 ) | 73 (43.71%) | 1 (50.00%) |  |
| Weak | 67 (39.64 ) | 66 (39.52%) | 1 (50.00%) |  |

Table . Biofilm formation ability of K20-positive and K20-negative *K. pneumoniae* isolates causing abscesses.

|  | **ALL**  ***N=169 (%)*** | **K20-negative**  ***N=166 (%)*** | **K20-positive**  ***N=3 (%)*** | **p.overall** |
| --- | --- | --- | --- | --- |
| result |  |  |  | 0.211 |
| Strong | 28 (16.57 ) | 27 (16.27%) | 1 (33.33%) |  |
| Medium | 74 (43.79 ) | 74 (44.58%) | 0 (0.00%) |  |
| Weak | 67 (39.64 ) | 65 (39.16%) | 2 (66.67%) |  |

**Table .** Biofilm formation ability of K54-positive and K54-negative *K. pneumoniae* isolates causing abscesses.

|  | **ALL**  ***N=169 (%)*** | **K54-negative**  ***N=167 (%)*** | **K54-positive**  ***N=2 (%)*** | **p.overall** |
| --- | --- | --- | --- | --- |
| result |  |  |  | 0.305 |
| Strong | 28 (16.57 ) | 27 (16.17%) | 1 (50.00%) |  |
| Medium | 74 (43.79 ) | 73 (43.71%) | 1 (50.00%) |  |
| Weak | 67 (39.64 ) | 67 (40.12%) | 0 (0.00%) |  |

Table . Biofilm formation ability of K57-positive and K57-negative *K. pneumoniae* isolates causing abscesses.

|  | **ALL**  ***N=169 (%)*** | **K57-negative**  ***N=164(%)*** | **K57-positive**  ***N=5(%)*** | **p.overall** |
| --- | --- | --- | --- | --- |
| result |  |  |  | 1.000 |
| Strong | 28 (16.57 ) | 27 (16.46%) | 1 (20.00%) |  |
| Medium | 74 (43.79 ) | 72 (43.90%) | 2 (40.00%) |  |
| Weak | 67 (39.64 ) | 65 (39.63%) | 2 (40.00%) |  |

Table . Biofilm formation ability of K64-positive and K64-negative *K. pneumoniae* isolates causing abscesses.

|  | **ALL**  ***N=169 (%)*** | **K64-negative**  ***N=150(%)*** | **K64-positive**  ***N=19(%)*** | **p.overall** |
| --- | --- | --- | --- | --- |
| result |  |  |  | 0.027 |
| Strong | 28 (16.57 ) | 28 (18.67%) | 0 (0.00%) |  |
| Medium | 74 (43.79 ) | 67 (44.67%) | 7 (36.84%) |  |
| Weak | 67 (39.64 ) | 55 (36.67%) | 12 (63.16%) |  |
